# Protective Effects and Mechanisms of Yinchen Linggui Zhugan Decoction in HFD-Induced Nonalcoholic Fatty Liver Disease Rats Based on Network Pharmacology and Experimental Verification

**DOI:** 10.3389/fphar.2022.908128

**Published:** 2022-06-02

**Authors:** Hui Jiang, Tangyou Mao, Yuyue Liu, Xiang Tan, Zhongmei Sun, Yuan Cheng, Xiao Han, Yang Zhang, Jiali Wang, Lei Shi, Yi Guo, Junxiang Li, Haixiao Han

**Affiliations:** ^1^ School of Graduate, Beijing University of Chinese Medicine, Beijing, China; ^2^ Dongfang Hospital, Beijing University of Chinese Medicine, Beijing, China; ^3^ Shenzhen Traditional Chinese Medicine Hospital, Shenzhen, China; ^4^ Dongzhimen Hospital, Beijing University of Chinese Medicine, Beijing, China

**Keywords:** Yinchen Linggui Zhugan decoction, nonalcoholic fatty liver disease, traditional Chinese medicine, experimental verification, network pharmacology, molecular docking

## Abstract

Nonalcoholic fatty liver disease (NAFLD) is a common chronic liver disease, characterized by excessive accumulation of hepatocyte fat. However, there is no exact and effective pharmacotherapy for NAFLD. Yinchen linggui zhugan decoction (YLZD) has been widely used to treat NAFLD. Nevertheless, its pharmacological and molecular mechanisms have not been clearly elucidated. This study was carried out to investigate the active components of YLZD and explore its potential mechanisms for treating NAFLD by network pharmacology and experimental verification. The results showed that a total of 120 active components of YLZD and 365 targets were retrieved through databases, and the main active ingredients of YLZD consisted of chlorogenic acid, emodin, aloe-emodin, rhein, and geniposide. KEGG enrichment analysis revealed fundamental roles of TNF, PI3K/AKT, HIF-1α, and insulin resistance signaling pathways in the treatment of NAFLD by YLZD. Moreover, our experimental verification results showed that YLZD improved the liver pathological and cholesterol level, and reduced the expressions of TNF-α, IL-1β, IL-6, NF-κB, CCL2, and CXCL10 in NAFLD rats, which all belonged to TNF signaling pathway. The molecular docking confirmed the correlation between the four core components (chlorogenic acid, emodin, rhein, and geniposide) and key factors (TNF-α, IL-6, and NF-κB) in TNF signaling pathway. In conclusion, the present study systematically clarified the protective mechanisms of YLZD against NAFLD through targeting the TNF signaling pathway, and provided new ideas for the drug research of this disease.

## Introduction

Nonalcoholic fatty liver disease (NAFLD) is characterized by excessive accumulation of hepatocyte fat (Pierantonelli and Svegliati-Baroni, 2019), which includes nonalcoholic fatty liver (NAFL), nonalcoholic steatohepatitis (NASH) and cirrhosis, and can even evolve into liver failure and hepatocellular carcinoma (HCC) without effective treatment (Friedman et al., 2018; Tokushige et al., 2021). Nearly a quarter of the world’s population is affected by NAFLD, and the prevalence together with incidence rate of NAFLD worldwide is increasing every year (Younossi et al., 2016; Peng et al., 2020). NAFLD has become a major cause of chronic liver disease, with a prevalence of 26%–45% according to epidemiological surveys (Anstee et al., 2019; Wu et al., 2020). At present, no pharmacotherapy has been approved for NAFLD treatment. Therefore, various medical fields are exploring new methods for the treatment of NAFLD.

Traditional Chinese Medicine (TCM) has been found to be effective for treating NAFLD in China and some other Asian countries (Wang et al., 2020; Dai et al., 2021; Fan et al., 2021). Previous studies have proved that TCM could improve metabolic diseases, especially NAFLD and NASH. Wei et al. (2021) reported that Sinisan protect against NAFLD by reducing hyperlipidemia, liver steatosis, and inflammation. Nie et al., (2020), Wu et al., (2021) demonstrated the effectiveness of Chaihu Shugan powder and ZeXie Decoction in the treatment of NAFLD through network pharmacology and experimental verification. Yinchen linggui zhugan decoction (YLZD) is the combination of Yinchenhao decoction and Linggui zhugan decoction, which is composed of the following seven well-established Chinese herbs: *Artemisiae scopariae* (“Yinchen” in Chinese), *Gardeniae fructus* (“Zhizi” in Chinese), *Radix rhei et rhizome* (“Dahuang” in Chinese), *Poria* (“Fuling” in Chinese), *Cinnamomi ramulus* (“Guizhi” in Chinese), *Atractylodes macrocephala koidz.* (“Baizhu” in Chinese), and *Licorice* (“Gancao” in Chinese). Our previous study showed that YLZD improved oxidative stress and lipid metabolism in HFD-induced NAFLD rats (Guo et al., 2017), but its pharmacological and molecular mechanisms still have not been clearly elucidated, which is mainly because YLZD, as a member of TCM, has the multi-component, multi-target and multi-pathway therapeutic characteristics.

**FIGURE 1:**
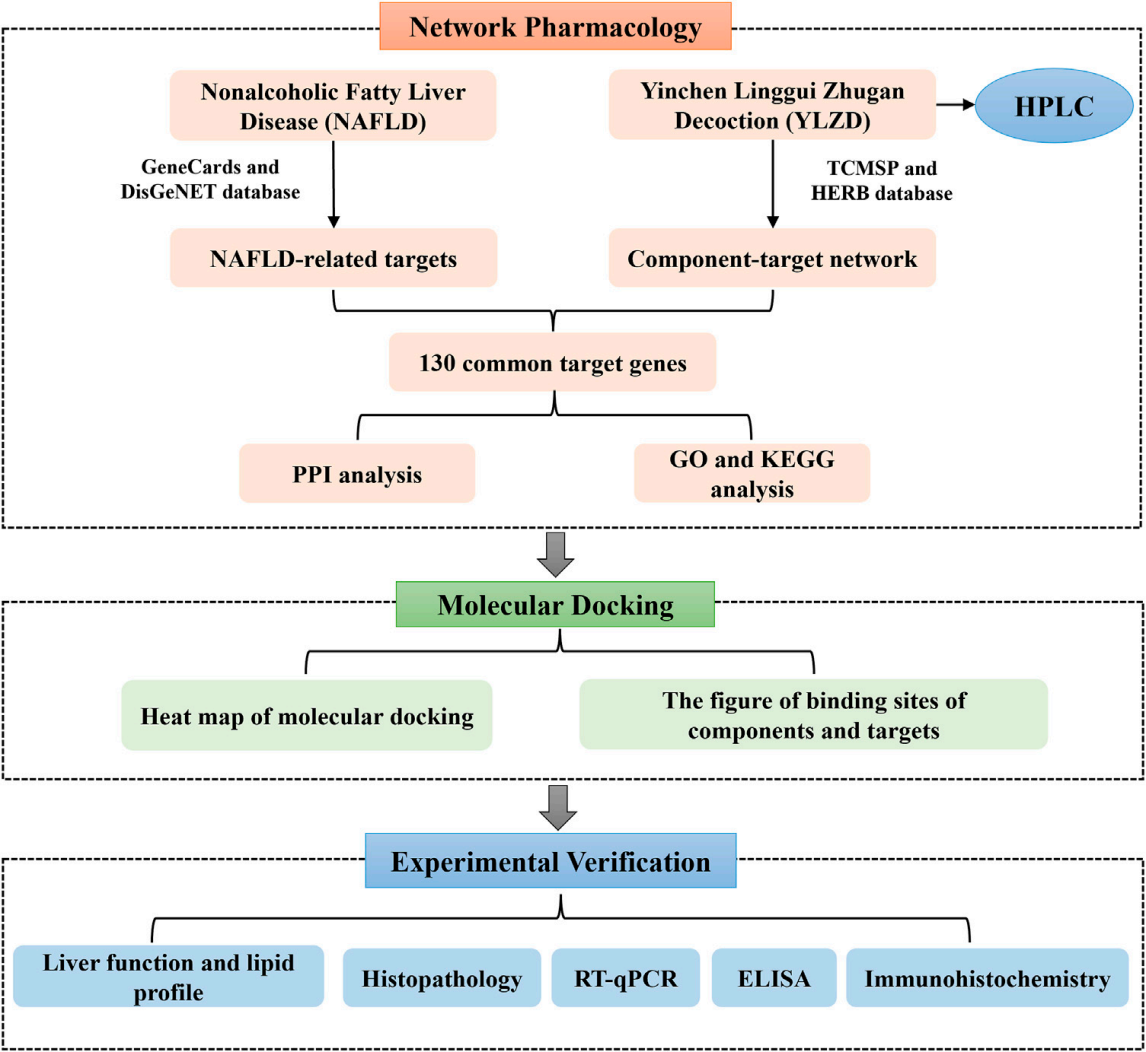
Main Workflow of this study.

Network pharmacology, as a bridge between TCM and modern medicine, is a new interdisciplinary approach (Hopkins, 2008; Wang X et al., 2021). Network pharmacology reveals the action mechanism of drugs on diseases at the overall level, explains the action mechanism of TCM and predicts potential targets (Li and Zhang, 2013). Molecular docking can predict the binding mode between receptors and ligands through simulation, and has been mainly applied in recent years for drug design and screening on the basis of receptors and ligands structures (Liu et al., 2021). Therefore, this study aimed at predicting the YLZD components, targets and signaling pathways against NAFLD from a network pharmacology aspect. A NAFLD animal model induced by HFD was established to investigate the efficacy of YLZD and to further explore the action mechanisms *in vivo*. Furthermore, a molecular docking technology was used for verification. The workflow of this investigation is shown in Figure 1. Significantly, the efficacy of YLZD against NAFLD is first thoroughly investigated through network pharmacology, molecular docking, and experimental verification in this research. The study may provide a theoretical foundation for further studies and reasonable clinical applications of YLZD against NAFLD.

## Materials and Methods

### Obtaining the YLZD Ingredients, Potential Targets and NAFLD-Related Gene Set

The Traditional Chinese Medicine Systems Pharmacology (TCMSP) Database (http://tcmspw.com/tcmsp.php) (Ru et al., 2014) was employed to collect the active components in YLZD. Oral bioavailability (OB) ≥30% and drug likeness (DL) ≥0.18 were set as screening conditions (Leeson and Springthorpe, 2007; Xu et al., 2012). In addition, the literatures were reviewed to identify the components in YLZD that were eliminated due to the above screening conditions but have potential research value for NAFLD, and the active components without targets were removed.

Furthermore, the TCMSP and HERB (http://herb.ac.cn/) (Fang et al., 2021) databases were used to find all potential targets of YLZD. The collected targets were corrected by the UniProt database (https://www.uniprot.org) (UniProt Consortium, 2021) with the protein genus set to “*Homo sapiens* (Human),” and Cytoscape (v 3.6.1) software was used to construct the active component-target network. NAFLD-related potential targets were collected from the GeneCards (https://www.genecards.org/) (Stelzer et al., 2011) and DisGeNET (http://disgenet.org/) (Piñero et al., 2020) databases. The overlapped target gene data associated with YLZD active components and NAFLD were retained as the primary targets for further analysis after the removal of duplicate gene information.

### Construction of Protein-Protein Interaction Network

The YLZD action targets were intersected with NAFLD targets using a Venn diagram (http://bioinformatics.psb.ugent.be/webtools/Venn/) to obtain the anti-NAFLD action targets of YLZD. Next, these intersecting targets were uploaded to the STRING database (https://string-db.org/) (Von Mering et al., 2005) for obtaining Protein-Protein Interaction (PPI) information. Protein interactions with confidence scores of 0.9 or higher were exported in TSV format. PPI networks were visualized by Cytoscape.

### Enrichment Analysis of Gene Ontology and Kyoto Encyclopedia Genes Genomes Pathways

The DAVID database (https://david.ncifcrf.gov/) (Jiao et al., 2012) was applied for Gene Ontology (GO) terms and Kyoto Encyclopedia Genes Genomes (KEGG) enrichment analysis. The GO and KEGG pathways were considered statistically significant at *p* < 0.01. Next, the pathways in the top 10 or 20 of the above analyses were visualized *via* Bioinformatics cloud platform (http://www.bioinformatics.com.cn/) to generate bubble plots. Finally, the component-target-pathway-disease network was established by Cytoscape to further illustrate the action mechanisms and targets of YLZD for treating the NAFLD.

### Preparation and High Performance Liquid Chromatography Analysis of YLZD

YLZD was provided by the Pharmacy Department of Dongfang Hospital, Beijing University of Chinese Medicine (Beijing, China) and consisted of the following herbs: 1.8 g *Artemisiae scopariae*, 0.9 g *G*. *fructus*, 1.2 g *Radix rhei et rhizome*, 2.4 g *Poria*, 0.45 g *Cinnamomi ramulus*, 1.2 g *Atractylodes macrocephala koidz*, and 1.2 g *Licorice*. All herbal decoction pieces were decocted twice after soaking, and the solid-liquid separation was carried out while hot. Concentration was performed at a reduced pressure and a temperature lower than 65°C and then freezing method was used for drying. For quality assurance, the procedure was carried out by Beijing Tcmages Pharmaceutical Co., Ltd. (Beijing, China) in accordance with the Good Manufacturing Practice for Drugs. For High Performance Liquid Chromatography (HPLC) analysis, standards of chlorogenic acid, geniposide, isochlorogenic acid A, isochlorogenic acid B, isochlorogenic acid C, aloe-emodin, rhein, glycyrrhizic acid, and emodin were purchased from Chengdu Pufei De Biotech Co., Ltd. (Chengdu, China). Then, a Waters 2695 HPLC system and a Hypersil ODS-2 Column (5 μm, 4.6 mm × 250 mm) were employed for the major YLZD components detecting. The column temperature was adjusted to 30°C, the flow rate was set as 1.0 ml/min and detection wavelength was 203 nm. Acetonitrile and 0.1% phosphoric acid solution were used as mobile phase A and B, respectively. The following gradient conditions were applied: 0.0∼60 min with 10%–50% A and 90%–50% B; 60–80 min with 50%–90% A and 50%–10% B; 80–80.1 min with 90%–10% A and 10%–90% B; 80.1–85 min with 10% A and 90% B.

### Molecular Docking Validation

The 3D structures of the target proteins were obtained from the RCSB PDB database (https://www.rcsb.org/) (Goodsell et al., 2020), and the active components were got from PubChem database (https://pubchem.ncbi.nlm.nih.gov/) (Wang et al., 2012) and were used as ligands. Water molecules removal, nonpolar hydrogen adding and the calculation of the affinities between these proteins and ligands were completed by AutoDockTools (v 1.5.7) software (El-Hachem et al., 2017). The docking results were visualized using the PyMOL (v 2.3.0) software (Baugh et al., 2011).

### Experimental Animals

Six-week-old male Sprague-Dawley (SD) rats (weighing 180 ± 20 g) were bought from Beijing Vital River Laboratory Animal Technology Co., Ltd. (Beijing, China). The experimental animals were all housed in a controlled environment with temperature of 22 ± 2°C, humidity of 50∼60% and a 12/12 h light/dark cycle. The rats were free to sterile water and standard laboratory chow during the adaptive feeding for 7 days. The experimental process was conducted strictly following the guidelines for the management and the use of laboratory animals of Animal Ethics Committee of Beijing University of Chinese Medicine (No. BUCM-4-2020102104-4162).

### Induction of NAFLD and Drug Administration

Thirty SD rats were randomly divided into a negative control group (NC, *n* = 6), model group (*n* = 24). The NC group rats were fed with standard laboratory feed for 10 weeks, followed by gavage with sterile distilled water (1.0 ml/kg/day) for 4 weeks. The model group rats were fed with HFD (88% basic diet, 10% lard, and 2% cholesterol) purchased by Beijing Keao Xieli Feed Co., Ltd. (Beijing, China) for 10 weeks to induce NAFLD. After successful model making, the rats were randomly divided into the HFD group (*n* = 6), YLZD low-dose group (YLZD-L, *n* = 6), medium-dose group (YLZD-M, *n* = 6), and high-dose group (YLZD-H, *n* = 6). During the 4-weeks intervention period, the HFD group rats were orally administered sterile distilled water (1.0 ml/kg/day) and the intervention group was instilled with different concentrations of YLZD (YLZD-L, 3.465 g/kg/day; YLZD-M, 6.93 g/kg/day; and YLZD-H, 13.86 g/kg/day). The equivalent dose for animals was obtained by calculating the equivalent amount of body surface area ratio between experimental animal and human. The equivalent dose of YLZD administered in this study was 6.93 g/kg, which is the medium-dose. The low-dose (3.465 g/kg) was 1/2 times of the medium-dose and the high-dose (13.86 g/kg) was twice of the medium-dose. The behavioral activities and fur status of rats were observed every day, and the weight together with food intake of rats were monitored every week. After 4 weeks’ treatment, the rats were anesthetized and sacrificed to collect serum and liver tissue samples.

### Blood Chemistry Analysis for Liver Function and Lipid Profiles

Rats were anesthetized after 12 h of overnight fasting and blood samples were collected. The blood was centrifuged at a low rotation rate of 3,000 rpm, 15 min at 4°C, and the supernatant was immediately aspirated. Then an AU480 Automatic Biochemical Analyzer (Beckman Coulter, Brea, CA, United States) was used to analyze the contents of alanine aminotransferase (ALT), aspartate aminotransferase (AST), total cholesterol (TC) and triglyceride (TG).

### Histological Analysis

Fresh liver tissues were fixed in 4% paraformaldehyde fixative for 24 h. After several steps such as ethanol dehydration, xylene transparency, and paraffin embedding, the sections were produced using a microtome and then they were stained with hematoxylin and eosin (H and E). In addition, the frozen liver tissues were sliced and mounted on glass slides to recover to room temperature and soaked in distilled water for 5 min and 60% isopropanol for 2 min. Then they were stained with Oil Red O (ORO) dye for 10 min and fully washed with distilled water. The histopathological changes in the H&E and ORO-stained slides were characterized by light microscopy.

### RNA Extraction and Quantitative Reverse Transcription PCR (RT-qPCR)

The total RNA extraction was carried out after the liver tissues homogenization by Trizol reagent [TIANGEN BIOTE (BEIJING) Co., Ltd.]. The concentration and purity of RNA were determined *via* a NanoDrop^®^ ND-2000 (Therno scientific). Ten microliters of RNA were extracted from each rat, PrimeScript™ RT reagent Kit with gDNA Eraser [Takara Biomedical Technology (Beijing) Co., Ltd.] was performed to reverse transcription. Then, the RT-qPCR was performed on the ABI7500 fast system (Applied Biosystems). The relative mRNA expression was calculated by the 2^−ΔΔCT^ method. The primers of tumor necrosis factor-α (TNF-α), interleukin-6 (IL-6), interleukin-1β (IL-1β), nuclear factor kappa-B (NF-κB), C-C motif chemokine 2 (CCL2), and C-X-C motif chemokine 10 (CXCL10) were designed and synthesized by Invitrogen. The sequences of the primers are given in Table 1.

**TABLE 1:** The sequences of RT-qPCR primers.

Genes	Forward primer	Reverse primer
*TNF-α*	TGA​ACT​TCG​GGG​TGA​TCG​GT	GGC​TAC​GGG​CTT​GTC​ACT​CG
*IL-6*	GAT​TGT​ATG​AAC​AGC​GAT​GAT​GC	AGA​AAC​GGA​ACT​CCA​GAA​GAC​C
*IL-1β*	GGG​ATG​ATG​ACG​ACC​TGC​TA	CCA​CTT​GTT​GGC​TTA​TGT​TCT​G
*NF-κB*	ACG​ATC​TGT​TTC​CCC​TCA​TC	TGC​TTC​TCT​CCC​CAG​GAA​TA
*CCL2*	GTG​TCC​CAA​AGA​AGC​TGT​AGT​ATT​T	TGC​TGA​AGT​CCT​TAG​GGT​TGA​T
*CXCL10*	GCA​CCT​GCA​TCG​ACT​TCA​T	TCTTTGGCTCACCGCTTT
*GAPDH*	AGA​GGG​AAA​TCG​TGC​GTG​A	CAT​TGC​CGA​TAG​TGA​TGA​CCT

### Enzyme-Linked Immunosorbent Assay

The collected liver tissues were washed 2 to 3 times with physiological saline to remove blood and to strip the fat from the connective tissue on the surface. 1 g liver tissues were accurately weighed, cut up and homogenized with 9 ml phosphate buffer (0.01 mol/L) on ice and then the mixtures were centrifuged at 3,000 rpm for 15 min at 4°C. The supernatant was removed and stored in a refrigerator at −20°C. The levels of liver TNF-α (No. YJ432890), IL-6 (No. YJ052732), IL-1β (No. YJ435222), NF-κB (YJ093211), CCL2 (No. YJ097621), and CXCL10 (No. YJ654321) were detected through conventional double-sandwich Enzyme-Linked Immunosorbent Assay (ELISA). The above kits were purchased from Shanghai Enzyme-linked Biotechnology Co., Ltd. (Shanghai, China), and the procedures were operated strictly following the kit instructions.

### Immunohistochemistry (IHC)

Paraffin sections were routinely dewaxed and placed in EDTA antigen repair buffer (pH 9.0, No. G1203) for antigen retrieval. After washing in PBS (pH 7.4), the sections were placed in a 3% H_2_O_2_ solution and incubated for 25 min at room temperature in dark to block endogenous peroxidase. Then, non-specific binding sites on the sections were blocked off using 3% BSA, and the tissues were incubated with primary antibodies of TNF-α (1:200, No. GB11188), IL-6 (1:500, No. GB11117), and NF-κB p65 (1:300, No. 66535-1-IG) overnight at 4°C. After that, the sections were incubated with secondary antibody of HRP conjugated Goat Anti-Rabbit IgG (H + L) (1:200, No. GB23303) and Anti-Mouse IgG (H + L) (1:200, No. GB23301) at room temperature for 50 min. The antibodies used in the experiment were purchased from Wuhan Servicebio Technology Co., Ltd. (Wuhan, China) and Proteintech Group, Inc. (Wuhan, China). Finally, the expressions of TNF-α, IL-6 and NF-κB p65 in liver tissue were observed using light microscope after color development with DAB, re-staining with hematoxylin, dehydration with ethanol, and sealing with neutral glue. The Image Pro Plus 6.0 software (Media Cybernetics, Inc., Rockville, MD, United States) was adopted to analyze the IOD/Area (Jia et al., 2021).

### Statistical Analysis

The statistical analysis was carried out by Graphpad Prism 8.0, and the measurement results were expressed as mean ± standard error of the mean (SEM). One-way analysis of variance was adopted for the comparison among multiple groups, and a marked statistical difference was determined at *p* < 0.05.

## Results

### Active Components and Potential Targets of YLZD

**FIGURE 2:**
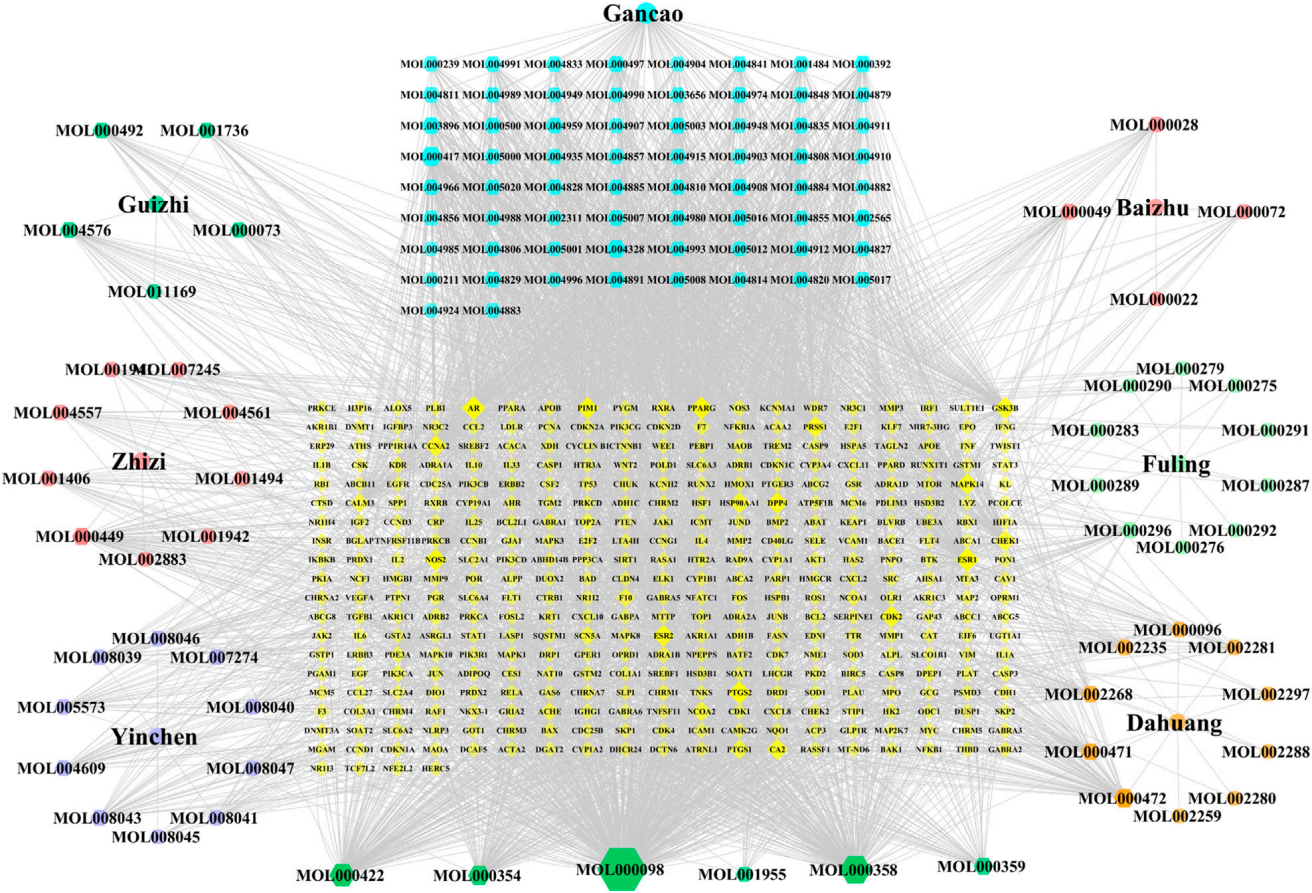
Component-target network of YLZD. The colorful hexagons show the major components of seven herbs. The gray lines indicate the interrelationships between nodes.

A total of 120 active components were obtained through TCMSP database and related literatures searching, and these ingredients were listed in detail in Supplementary Table S1. Then, the TCMSP and HERB databases were used to screen all potential targets of YLZD ingredients, and a total of 365 targets were obtained after the correction and removal of duplicate items by the UniProt database (Supplementary Table S2). After that, a component-target network containing 492 nodes with 3375 edges was constructed by Cytoscape, where yellow represents the drug potential targets and the other colors present the YLZD active components. As shown in Figure 2, quercetin, beta-sitosterol, kaempferol, isorhamnetin, calycosin, emodin, aloe-emodin, rhein, geniposide, chlorogenic acid, etc. may be the main active ingredients of YLZD.

### NAFLD-Related Targets

The term “nonalcoholic fatty liver disease” was used for searching in the GeneCards and DisGeNET databases. The median relevance score of the GeneCards database was calculated to be 20.63 twice, and 413 data points with relevance scores ≥20.63 were screened. A total of 140 data points with Score_gda ≥0.1 were screened from the DisGeNET database. A total of 487 potential disease targets associated with NAFLD were obtained after deduplication and correction using the UniProt database (Supplementary Table S3). The above targets were uploaded to the Bioinformatics cloud platform, and 130 overlapped targets were obtained (Supplementary Figure S1; Supplementary Table S4), which could be considered to be core targets in the anti-NAFLD pharmacological mechanisms of YLZD.

### Protein-Protein Interaction Network Construction and Analysis

**FIGURE 3:**
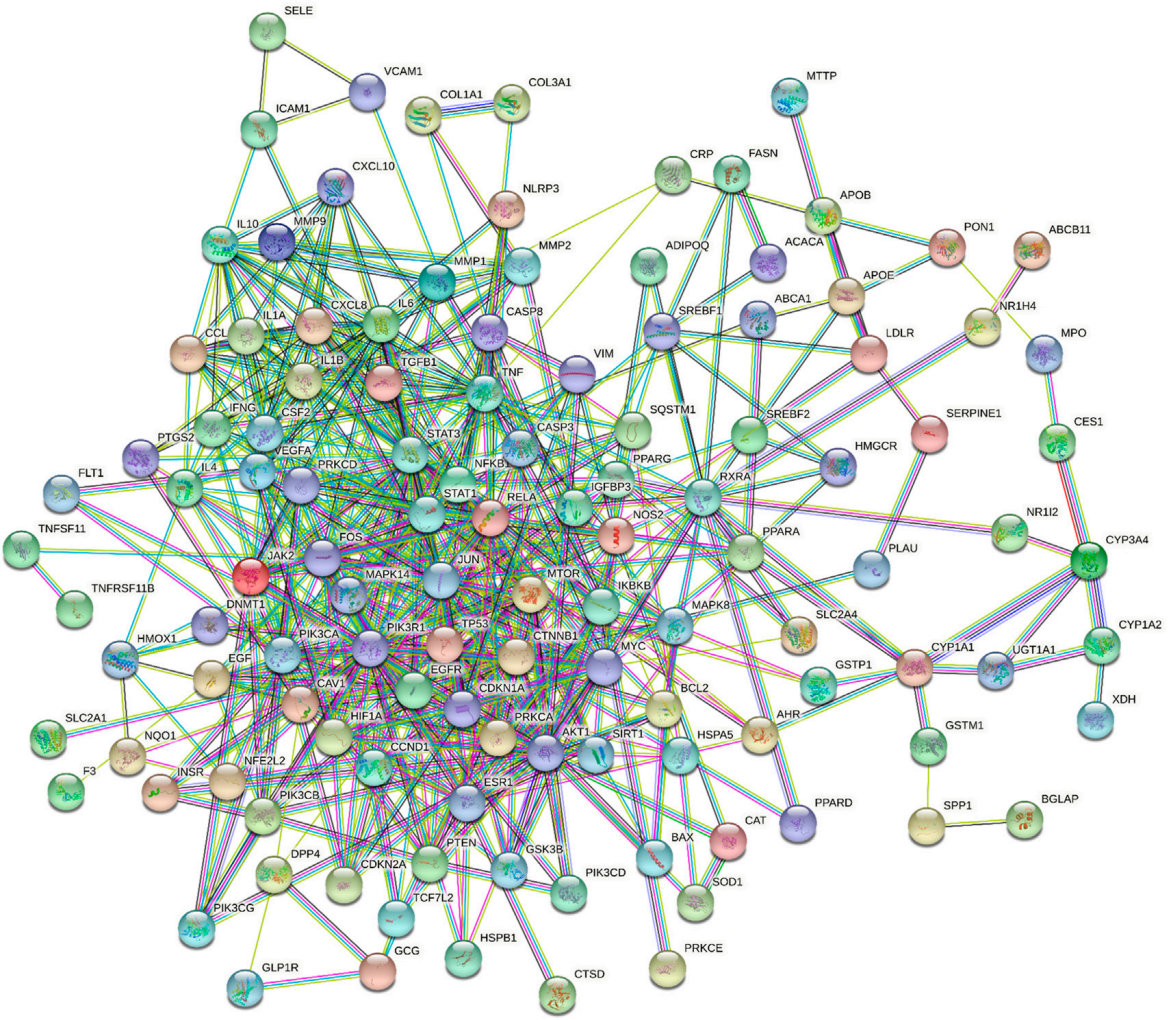
PPI network of YLZD for treating NAFLD.

The above 130 common targets were imported into the STRING database to obtain the protein interaction network. The PPI network in Figure 3 included 119 nodes and 563 edges. The network analyzer results exhibited that the average degree of nodes, the average betweenness centrality, the average closeness centrality and the average neighborhood connectivity were 9.462, 0.016, 0.363, and 15.217, respectively. There were 20 targets with values exceeding the average value (Supplementary Table S5). Then, the PPI network was visualized and displayed by Cytoscape (Supplementary Figure S2).

### GO and KEGG Functional Enrichment Analysis

**FIGURE 4:**
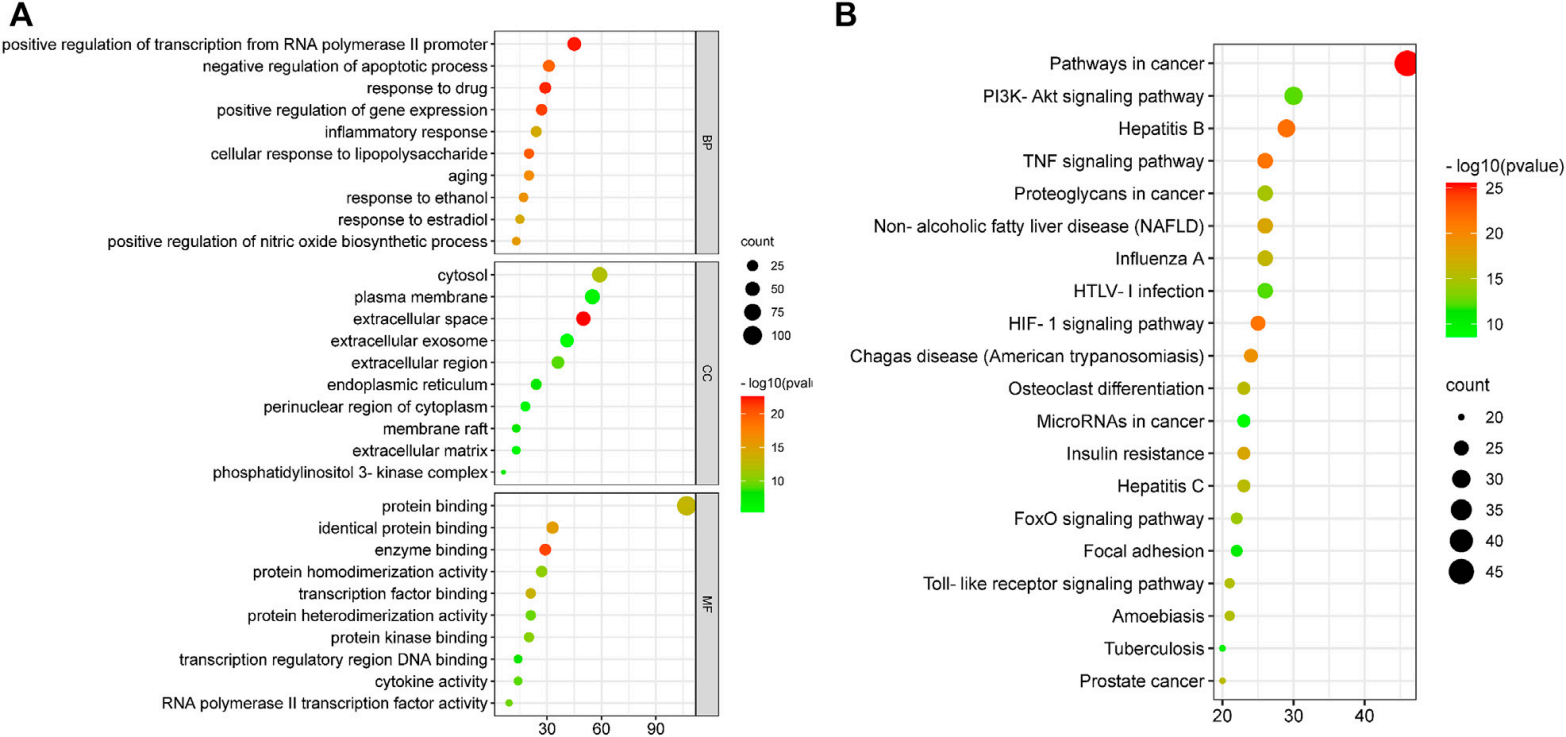
GO and KEGG functional analysis. **(A)** GO enrichment analysis for 130 core targets. **(B)** KEGG enrichment analyses for 130 core targets. The horizontal axis represents the proportion of enriched genes in entries, and the vertical axis represents each entry. The larger the number of enrichment targets, the larger the points; The higher the *p* value, the redder the color of the point.

The GO enrichment analysis mainly included biological process (BP), cellular component (CC) and molecular function (MF). A total of 261 biological entries were involved in the GO analysis of YLZD for NAFLD using counts >3, *p* < 0.01 and FDR <0.01 as screening conditions (Supplementary Table S6). Among them, 201 BPs were involved in the regulation of apoptosis, gene expression, NO production, inflammatory response, cellular response to lipopolysaccharide and transcription from the RNA polymerase II promoter; 27 CCs were involved in extracellular space, cytosol, endoplasmic reticulum, membrane raft, extracellular matrix and other aspects; 33 MFs were involved in protein binding, enzyme binding, transcription factor binding, cytokine activity and other respects. The top 10 related entries for BP, CC, and MF were displayed in bubble plots in Figure 4A.

DAVID database was employed to analyze the 130 overlapped targets between YLZD and NAFLD. 101 pathways (counts >3, FDR <0.01, *p* < 0.01) were obtained, and the results were given in Supplementary Table S7. The top 20 pathways were intuitively represented in a bubble plot, as shown in Figure 4B. KEGG enrichment results demonstrated that YLZD might against NAFLD by regulating the TNF, PI3K/AKT, HIF-1, insulin resistance (IR), and other signaling pathways. Furthermore, component-target-pathway-disease network was constructed for elucidating the interrelationship of components, targets, disease and the top 20 pathways, as shown in Supplementary Figure S3. In conclusion, these results illustrated that YLZD treats the NAFLD through a combination of multiple pathways, multiple targets, and overall cooperation.

### Major Components of YLZD by HPLC Analysis

**FIGURE 5:**
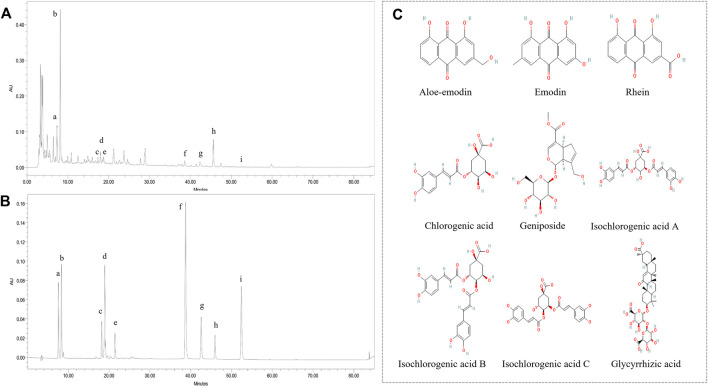
Major components of YLZD by HPLC analysis. **(A)** HPLC chromatograms of YLZD aqueous extract. **(B)** HPLC spectrum of the mixed standards. a) Chlorogenic acid; b) Geniposide; c) Isochlorogenic acid B; d) Isochlorogenic acid A; e) Isochlorogenic acid C; f) Aloe-emodin; g) Rhein; h) Glycyrrhizic acid; i) Emodin. **(C)** The chemical structures of the active components in YLZD.

To verify the presence of components screened out through network pharmacology in YLZD, HPLC was used to determine the components quantitatively. As shown in Figures 5A,B, chlorogenic acid, geniposide, aloe-emodin, rhein, and emodin were identified by referring to the corresponding standards. In the meantime, isochlorogenic acid B, isochlorogenic acid A, isochlorogenic acid C, and glycyrrhizic acid were also detected by HPLC because of their high contents. Figure 5C shows the chemical structures of the analytes and internal standards. The contents of chlorogenic acid, geniposide, isochlorogenic acid B, isochlorogenic acid A, isochlorogenic acid C, aloe-emodin, rhein, glycyrrhizic acid, and emodin were 2.8521, 11.8532, 0.6459, 0.3511, 0.8627, 0.0971, 0.1098, 2.9140, and 0.0088 (mg/g), respectively.

### Molecular Docking Results

**FIGURE 6:**
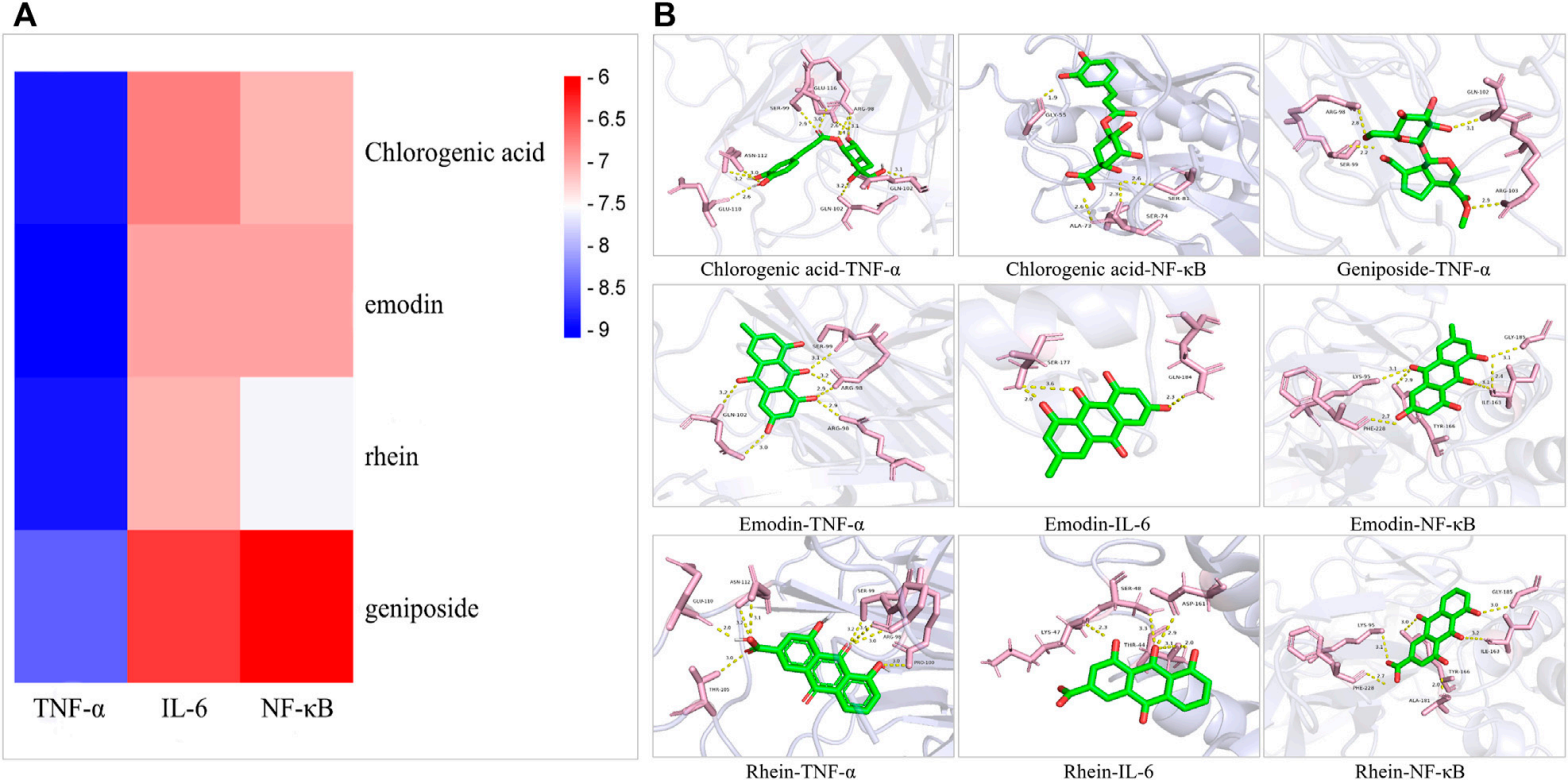
Molecular docking results. **(A)** Heat map for the binding energies of docked components within the active sites of tested targets. **(B)** Part of molecular docking results.

Four main active components (chlorogenic acid, emodin, rhein, and geniposide) and three core targets (TNF-α, IL-6, and NF-κB) on the TNF signaling pathway were used as ligands and receptors, respectively. The lower binding energy indicates a more stable binding conformation between the receptor and ligand (Li et al., 2021). A threshold value of −5.0 kcal/mol for binding energy was applied to determine the binding stability between receptor and ligand (Li et al., 2020). Thus, the ligand with lowest binding energy to the receptor was screened. The binding energy of key components of YLZD with core targets are shown in Figure 6A; Table 2, and part of the receptor-ligand interactions are displayed in Figure 6B.

**TABLE 2:** Binding energy of key components of YLZD with core targets.

Components	Binding energy (kcal/mol)		
	TNF-α (2E7A)	IL-6 (1IL6)	NF-κB (1SVC)
chlorogenic acid	−8.9	−6.8	−7.1
emodin	−9.1	−7.0	−7.0
rhein	−8.9	−7.1	−7.6
geniposide	−8.5	−6.4	−6.0

### YLZD Improved the General Status and Liver Injury Biochemical Indexes in NAFLD Rats

**FIGURE 7:**
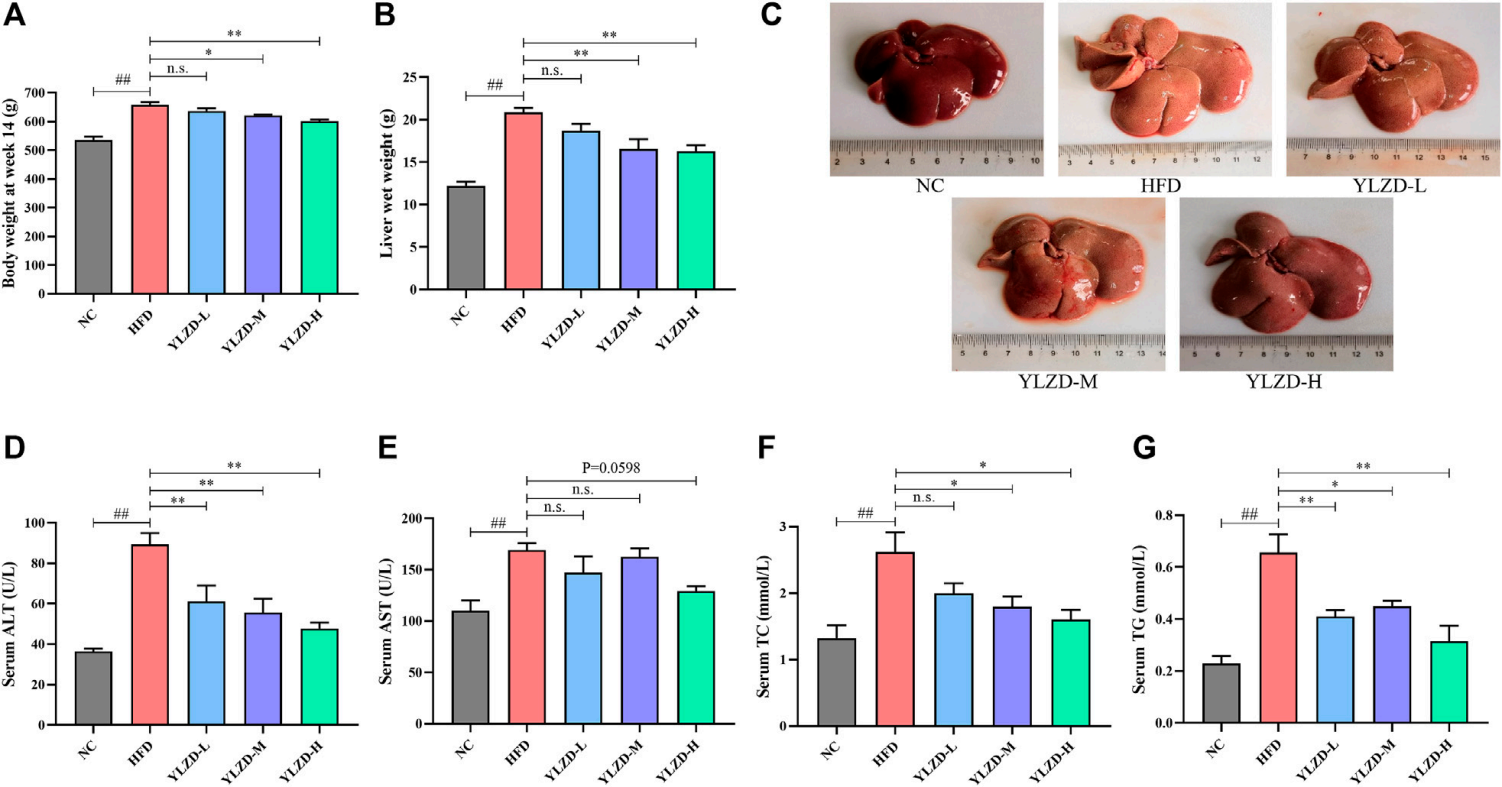
YLZD improved the general status and liver injury biochemical indexes in NAFLD rats. **(A)** Body weight at week14, **(B)** Liver wet weight, **(C)** Macroscopic view of liver. **(D–G)** Serum ALT, AST, TC, and TG. Data are expressed as mean ± SEM (*n* = 6 rats). ^##^ *p* < 0.01 versus the NC group, ^*^ *p* < 0.05 and ^**^ *p* < 0.01 versus the HFD group.

The network pharmacology analysis results were validated through the establishment and treatment of an HFD-induced NAFLD rat model with YLZD. NAFLD rats had notably higher body weights and liver wet weights at week 14 by comparison with the NC group (Figures 7A,B). An inspection of Figure 7C shows that the liver was diffusely enlarged with a pale or grayish yellow color, smooth surface, rounded edges and a greasy feeling. In contrast, the body weights at week 14 and liver wet weight of middle and high-dose YLZD treated rats were significantly reduced. Meanwhile, the liver color and texture in YLZD were improved to different degrees.

Moreover, the serum AST, ALT, TC, and TG levels were markedly increased in NAFLD rats compared with the NC group (Figures 7D–G). Notably, ALT and TG were reduced effectively by the YLZD, as depicted in Figures 7D–G,F shows that medium and high-dose YLZD decreased the serum TC. In addition, YLZD was also observed to reduce the level of serum AST to some extent, although the results were not statistically significantly different (Figure 7E). These findings indicated that YLZD improved general status and liver function, increased lipid excretion, and then alleviated hyperlipidemia of NAFLD rats.

### YLZD Ameliorated Pathological Damage in NAFLD Rats

**FIGURE 8:**
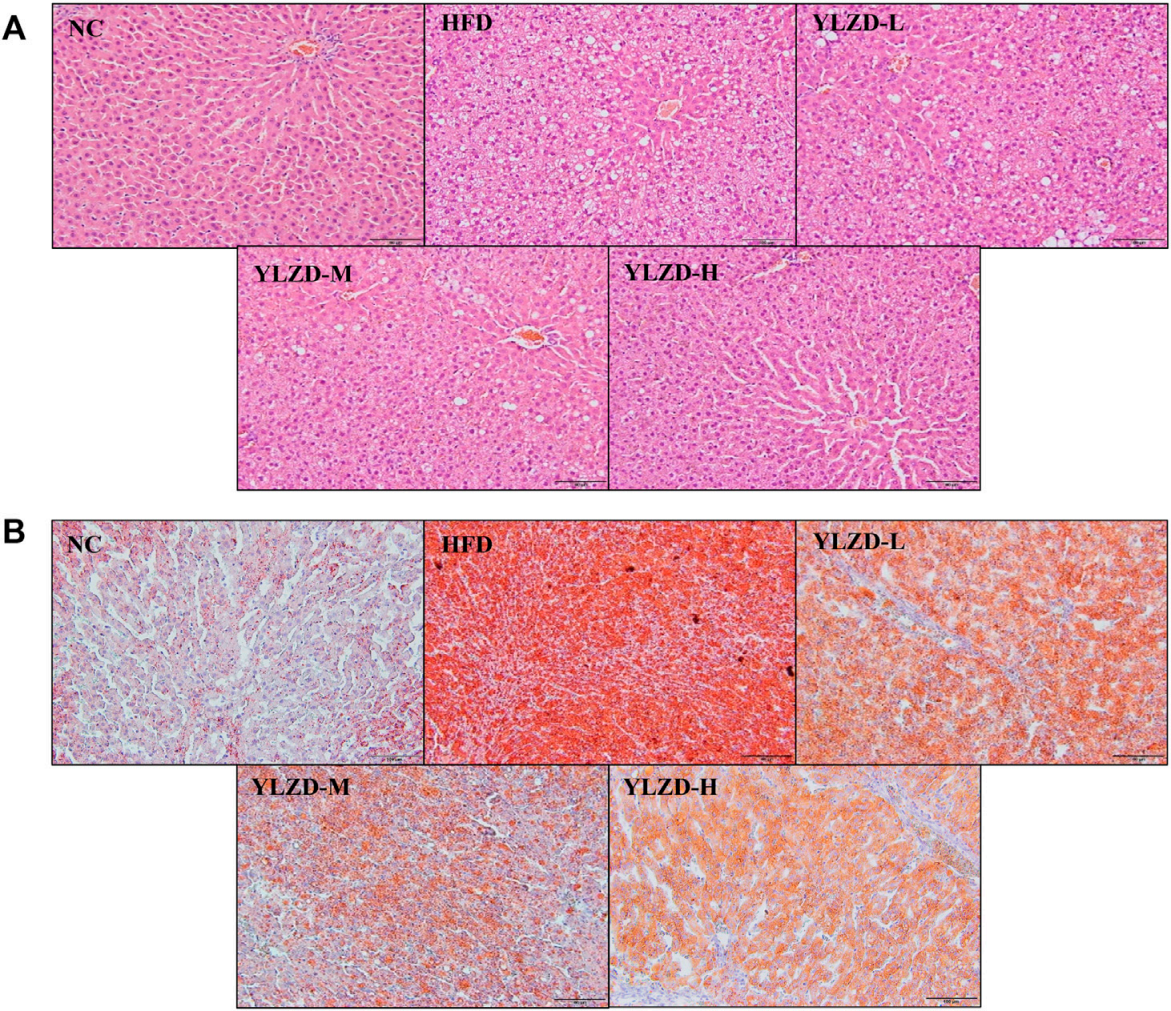
YLZD ameliorated pathological damage in NAFLD rats. **(A)** Representative photomicrographs of H&E staining (×200). **(B)** Representative photomicrographs of Oil Red O staining (×200).

H and E and Oil Red O staining of liver tissues in NC group showed a radial arrangement of hepatocytes, centered on the central vein, with clearly visible hepatic lobules and hepatic cords. No steatosis, necrosis or obvious inflammatory cell infiltration was observed (Figure 8). In the HFD group, the structure of liver plate was destroyed, and there were a large number of fat vacuoles, lipid droplet deposition and inflammatory cell infiltration in the liver. Compared with the HFD group, the dosing groups showed varying degrees of improvement in hepatocyte steatosis and inflammation in liver tissues, and the effects were more pronounced with increasing doses (Figures 8A,B). The histopathology results strongly demonstrate that YLZD attenuated hepatic pathological damage, reduced hepatic steatosis and inflammation in rats with NAFLD. An inspection demonstrated that high-dose (YLZD-H) can most effectively against NAFLD among the three different dosages. So, the YLZD high-dose group was selected for the further mechanistic research.

### YLZD Inhibited TNF Signaling Pathway-Related mRNA Expressions in NAFLD Rats

**FIGURE 9:**
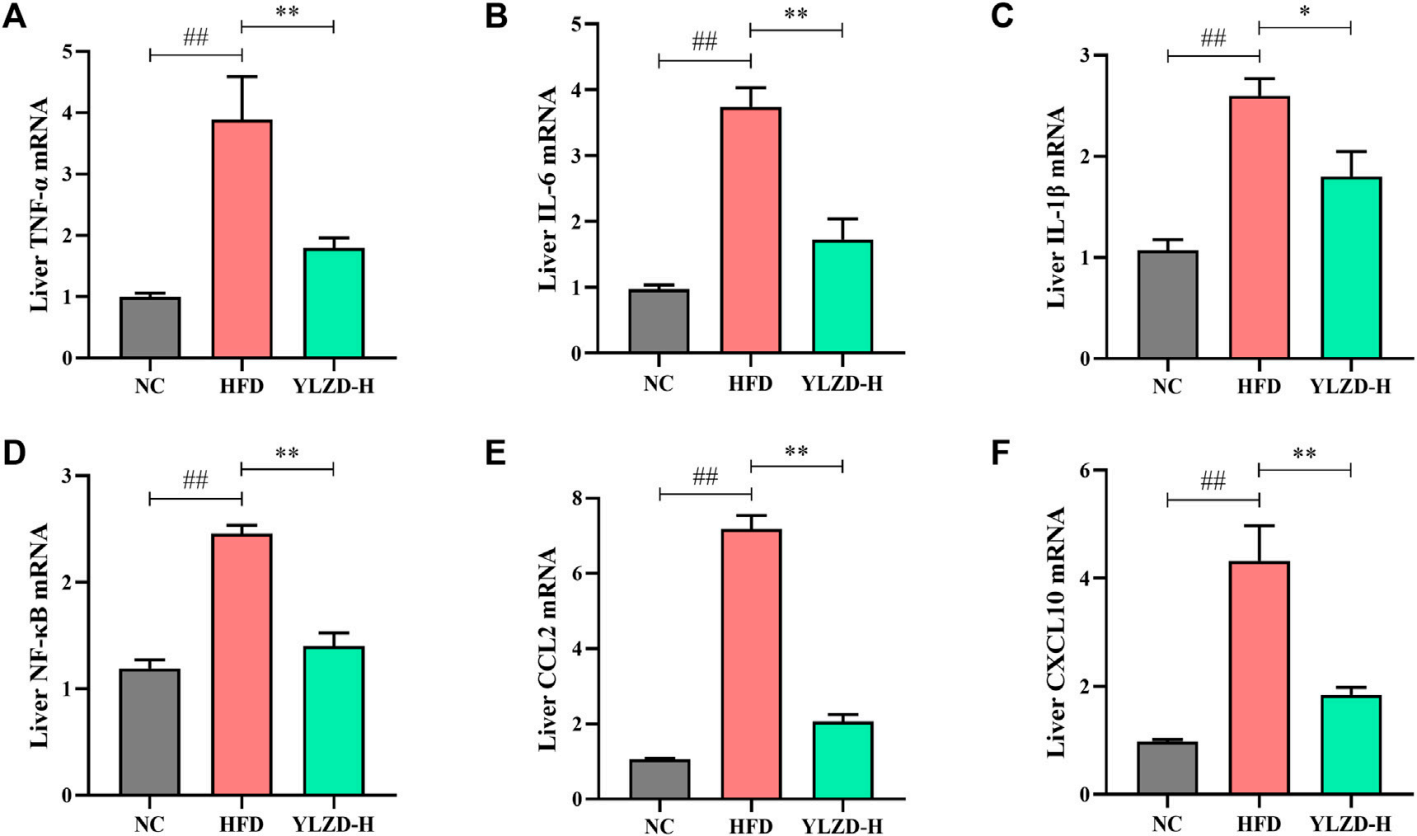
YLZD inhibited TNF signaling pathway-related mRNA expressions in rats. **(A–F)** RT-qPCR analysis for liver TNF-α, IL-6, IL-1β, NF-κB, CCL2, and CXCL10 relative mRNA expression. Data are expressed as mean ± SEM (*n* = 6 rats). ^##^ *p* < 0.01 versus the NC group, ^*^ *p* < 0.05 and ^**^ *p* < 0.01 versus the HFD group.

KEGG pathway enrichment analysis revealed that the identified candidate targets of YLZD were enriched in the TNF signaling pathway, which had a significant impact on the pathogenesis of NAFLD. Therefore, we focused on the exploration of TNF signaling pathway related factors expression for further verification. As shown in Figures 9A–F, *TNF-α*, *IL-6*, *IL-1β*, *NF-κB*, *CCL2*, and *CXCL10* gene expression levels were notably elevated in the liver of HFD-induced NAFLD rats, while YLZD treatment effectively inhibited the mRNA expression levels of these cytokines.

### YLZD Suppressed TNF Signaling Pathway-Related Protein Expressions in NAFLD Rats

**FIGURE 10:**
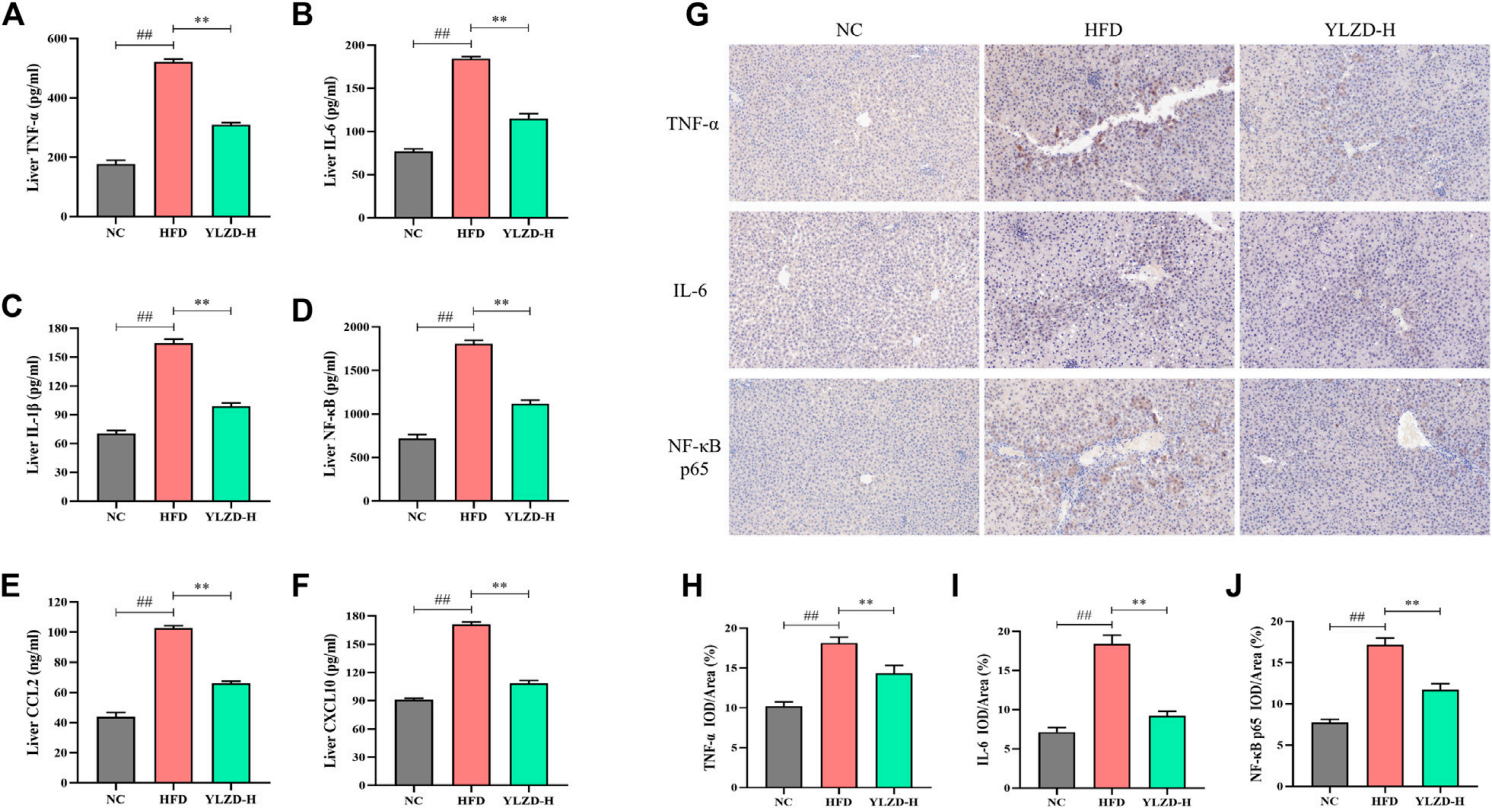
YLZD suppressed TNF signaling pathway-related protein expressions in NAFLD rats. **(A–F)** ELISA analysis of TNF-α, IL-6, IL-1β, NF-κB, CCL2, and CXCL10 protein expression. **(G)** Representative photomicrographs of immunohistochemistry staining (×200). **(H–J)** The effect of YLZD on the expression of TNF-α, IL-6, and NF-κB for treating NAFLD detected by immunohistochemistry staining (×200). Data are expressed as mean ± SEM (*n* = 6 rats). ^##^ *p* < 0.01 versus the NC group, ^**^ *p* < 0.01 versus the HFD group.

To further verify the expression of TNF signaling pathway-related proteins, the expression levels of TNF-α, IL-6, IL-1β, NF-κB, CCL2, and CXCL10 were measured by ELISA. As shown in Figures 10A–F, the protein levels of TNF-α, IL-6, IL-1β, NF-κB, CCL2, and CXCL10 were significantly increased in the HFD group compared with NC group. YLZD intervention remarkably reduced the expression level of the above proteins. Furthermore, IHC technique was also carried out to localize and quantify the expression of TNF-α, IL-6, and NF-κB p65. The results from Figure 10G showed that the positive expression of TNF-α, IL-6 and NF-κB p65 in the liver tissues of HFD-induced NAFLD rats had a larger range and dark staining of brown color compared with the NC group. In contrast, the staining range and depth of TNF-α, IL-6, and NF-κB p65 protein positive expressions were decreased to different degrees after YLZD intervention. IOD/Area analysis results (Figures 10H–J) suggested that the expression of TNF-α, IL-6 and NF-κB p65 proteins were significantly increased in HFD group compared with NC group, and these trends were significantly reversed after YLZD administration. In summary, all these findings declared that YLZD inhibited the expressions of TNF signaling pathway-related proteins and attenuated HFD-induced liver tissue injury and inflammatory response in rats with NAFLD.

## Discussion

NAFLD is a global public health problem with no U. S. Food and Drug Administration (FDA)-approved pharmacological therapies (Younossi et al., 2018). In recent years, TCM have received increased attention for treating NAFLD due to its clear pharmacological effects and few side effects (Yan et al., 2020). Previous study (Guo et al., 2017) has shown that YLZD had definite therapeutic effect on HFD-induced NAFLD rats. However, its multiple ingredients and multiple therapeutic mechanisms of anti-NAFLD required to be further clarified. Accordingly, a variety of ingredients and potential mechanisms of YLZD for treating NAFLD were investigated through a combination of network pharmacology, molecular docking and NAFLD animal model verification in the present study, aiming to provide an experimental basis for clinical applications.

Associations between the components and their corresponding potential targets were established by a component-target network. The results from network pharmacology approach and HPLC analysis demonstrated that emodin, aloe-emodin, rhein, geniposide, and chlorogenic acid may be the main active ingredients for YLZD in NAFLD treatment. Interestingly, previous investigations have revealed that these five ingredients were associated with NAFLD. Emodin, aloe-emodin and rhein are the main free anthraquinones of the *Radix rhei et rhizome* and have good anti-inflammatory, anti-tumor, cardiovascular and liver protective effects (Hu et al., 2020). For example, Jia et al. (2014) observed that emodin could reduce the secretion of TNF-α, IL-1β, and IL-6, inhibit the expression of leukocyte chemokine CCL2, improve the inflammatory response in HFD mice and suppress the transition from simple steatosis to NASH . Dou et al. (2019) and Quan et al. (2019) reported that aloe-emodin exerted anti-inflammatory effects by reducing the production of inflammatory factors (TNF-α and IL-6) through various pathways such as NF-κB and PI3K/AKT. Geniposide, the critical active ingredient of *G*. *fructus*, could alleviate liver injury by enhancing antioxidant defense system and inhibiting apoptotic signaling pathways (Kim et al., 2013). It has been reported that geniposide was conducive to protecting mice and cells from NAFLD-induced oxidative stress and inflammation through upregulating the nuclear factor erythroid-2 related factor 2 (Nrf2) and regulating the protein expression of AMPK/PI3K/mTOR signaling pathways (Shen et al., 2020). Chlorogenic acid has been confirmed to effectively improve acute and chronic liver injury *via* antioxidant and anti-inflammatory effects (Shi et al., 2021). Moreover, chlorogenic acid was able to improve IR, regulate glucolipid metabolism, gut microbiota and glucagon-like peptide 1 (GLP-1) (Ma et al., 2015; Shi et al., 2021). This illustrates that chlorogenic acid is a potential drug for the prevention and treatment of NAFLD. These evidences suggested that the YLZD ingredients might exhibit anti-steatosis and anti-inflammation effects on NAFLD. Unfortunately, the above researches reported the effects of a single active ingredient, therefore, we plan to explore two or more combinations of these ingredients in the future, which is more in line with the multi-component and multi-target mechanisms of TCM.

Most notably, we found that TNF signaling pathway was more closely associated with NAFLD pathogenesis by comparison with other pathways on the basis of the KEGG pathway enrichment analysis. It is well known that aberrant regulation of inflammation is a prominent driver in the progression of NAFLD, and persistent inflammation can lead NAFLD to evolve into cirrhosis, HCC, and end-stage liver disease (Wang H et al., 2021). Abnormalities in the TNF signaling pathway was central to the persistence of chronic liver inflammation. Researches have confirmed that TNF-α expression levels were increased in the liver of NASH patients, which may be involved in lipid-peroxidation and oxidative stress in different ways. This induced an inflammatory response in the liver and a release of other inflammatory factors (IL-1β and IL-6) that imbalanced the inflammatory response in host (Lopetuso et al., 2018). High TNF-α concentration reduces lipolysis in peripheral tissues, promotes TG synthesis and aggregation in hepatocytes and increases free fatty acids (FFA) in the liver. While FFA can cause mitochondrial insufficiency and hepatocyte damage by enhancing the toxicity of cytokines such as TNF-α (Yang et al., 2019). NF-κB has a pleiotropic regulatory function and can bind to a variety of promoters and participate in the regulation of various inflammatory genes (Chen et al., 2020). When inflammatory changes occur in hepatocytes, serum levels of TNF-α increase, which induces NF-κB activity and promotes liver inflammatory responses and the activated NF-κB can in turn inversely promotes TNF-α expression and causes fatty liver injury (Deng et al., 2018; Mitchell and Carmody, 2018). CCL2 and CXCL10 are the common pro-inflammatory chemokines. Studies have shown that elevated CCL2 expression in the liver recruited immune cells to flow into the liver, thereby maintaining chronic tissue inflammation (Kim et al., 2015). CXCL10 amplifies the effects of other pro-inflammatory cytokines including TNF-α, IL-6, L-1β, and CCL2, and is therefore a key pro-inflammatory factor (Xu et al., 2016). In addition, CXCL10 is critical in the pathogenesis of NASH by inducing inflammation, regulating lipogenesis and oxidative stress (Zhang et al., 2014). In the present study, we observed that YLZD reversed the expression levels of TNF-α, IL-6, IL-1β, and NF-κB in liver tissues of NAFLD rats and decreased the expression of inflammatory chemokines CCL2 and CXCL10. Taken together, the results suggest that YLZD may suppress the inflammatory response of NAFLD by targeting the TNF signaling pathway. In addition, the other action mechanisms of YLZD treating NAFLD will be explored in our following research.

In summary, the main active components, candidate targets and related signaling pathways of YLZD for NAFLD treatment were obtained through network pharmacology. We found that YLZD might exert a therapeutic effect on NAFLD by inhibiting the TNF signaling pathway *via* experimental verification in NAFLD model rats. These findings provide novel insights into the regulatory role of YLZD in the treatment of NAFLD and hold promise for herb-based complementary and alternative therapy.

## Data Availability

The original contribution presented in the study are included in the article/Supplementary Material, further inquiries can be directed to the corresponding authors.

## Supplementary Material

Supplementary Table S1 Information of ingredients and corresponding targets of YLZD.

Supplementary Table S2 Targets of bioactive components in YLZD retrieved from databases.

Supplementary Table S3 Information of NAFLD-related targets collected from GeneCards and DisGeNET database.

Supplementary Table S4 YLZD-NAFLD-related targets.

Supplementary Table S5 Selected 20 core targets.

Supplementary Table S6 GO enrichment analysis of YLZD-NAFLD genes.

Supplementary Table S7 KEGG enrichment analysis of YLZD-NAFLD genes.
