# Supplementary material for: Protective Effects and Mechanisms of Yinchen Linggui Zhugan Decoction in HFD-Induced Nonalcoholic Fatty Liver Disease Rats Based on Network Pharmacology and Experimental Verification
Source: Front Pharmacol. 2022 Jun 2;13:908128. doi: 10.3389/fphar.2022.908128 (PMC9202027; doi:10.3389/fphar.2022.908128)

Supplementary Figure

**Supplementary Figure 1.** Venn diagram of predicted targets of NAFLD and YLZD.


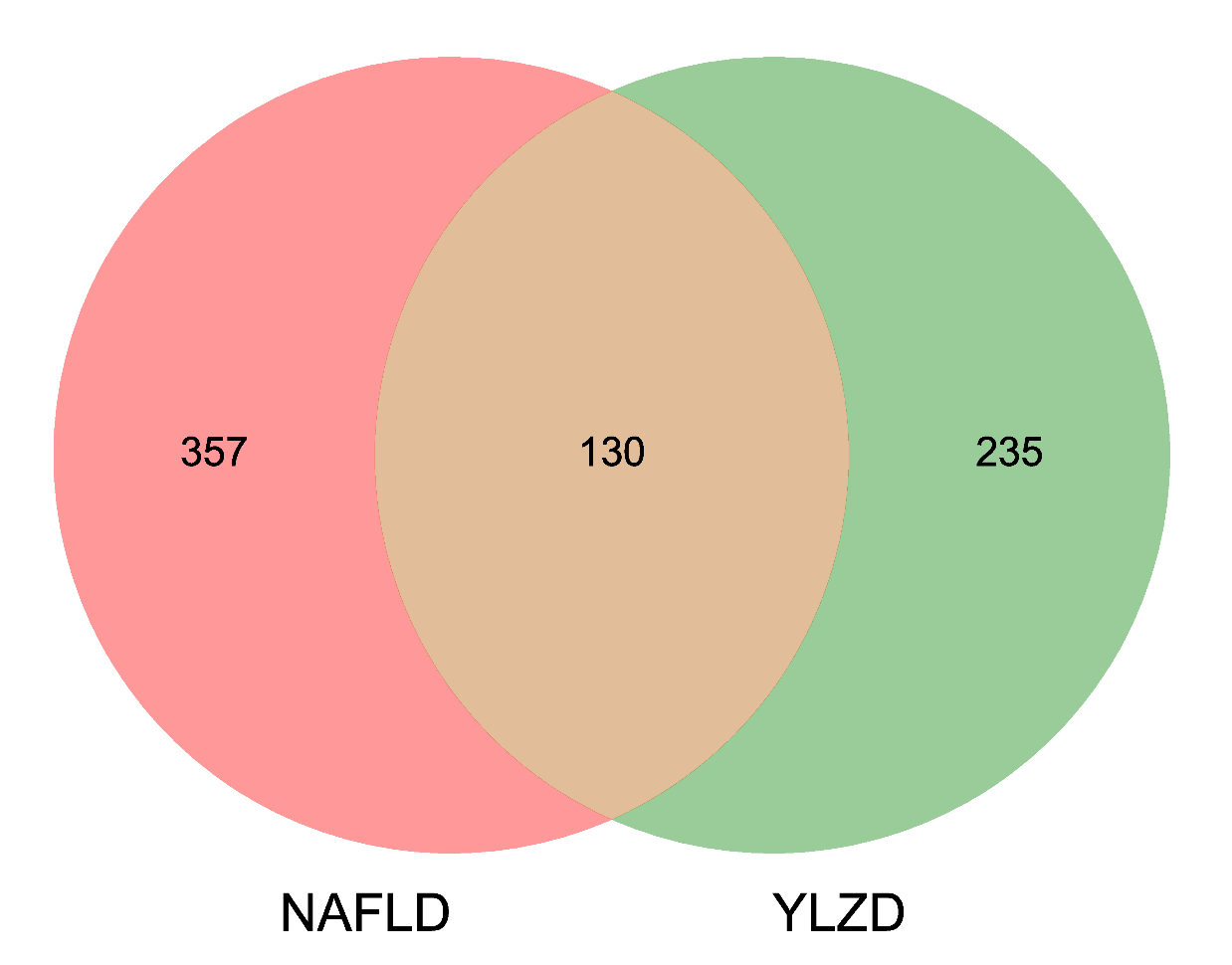


**Supplementary Figure 2.** Selected 20 core targets. Node size and color represented the size of degree value, and the larger and redder the node, the greater the corresponding degree value, and the higher the degree of interaction association of this protein with others.


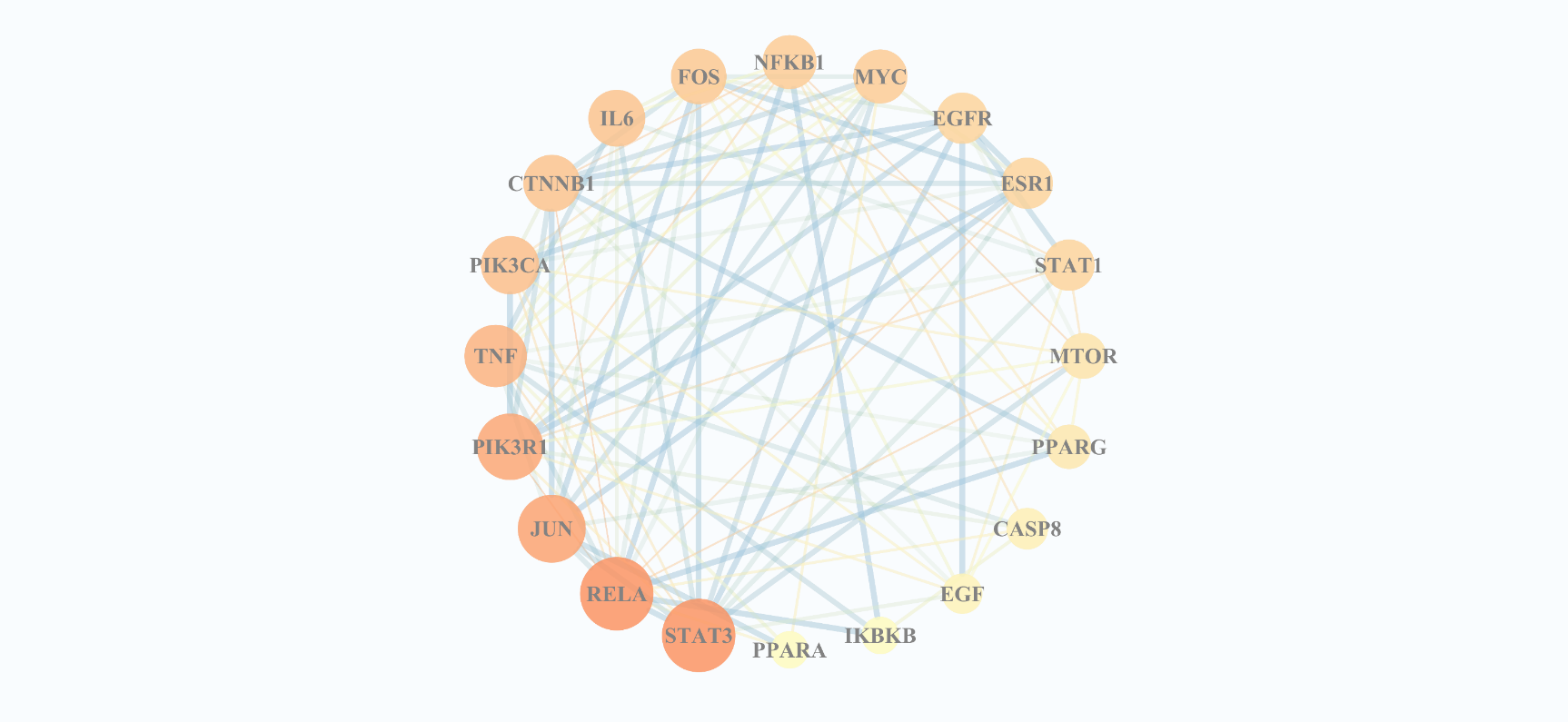


**Supplementary Figure 3.** Component-target-pathway-disease network. The blue diamonds stand for core genes, green triangles stand for pathways, red hexagons stand for main components of YLZD, and purple triangle stand for NAFLD. Gray lines indicate the interrelationships between nodes.


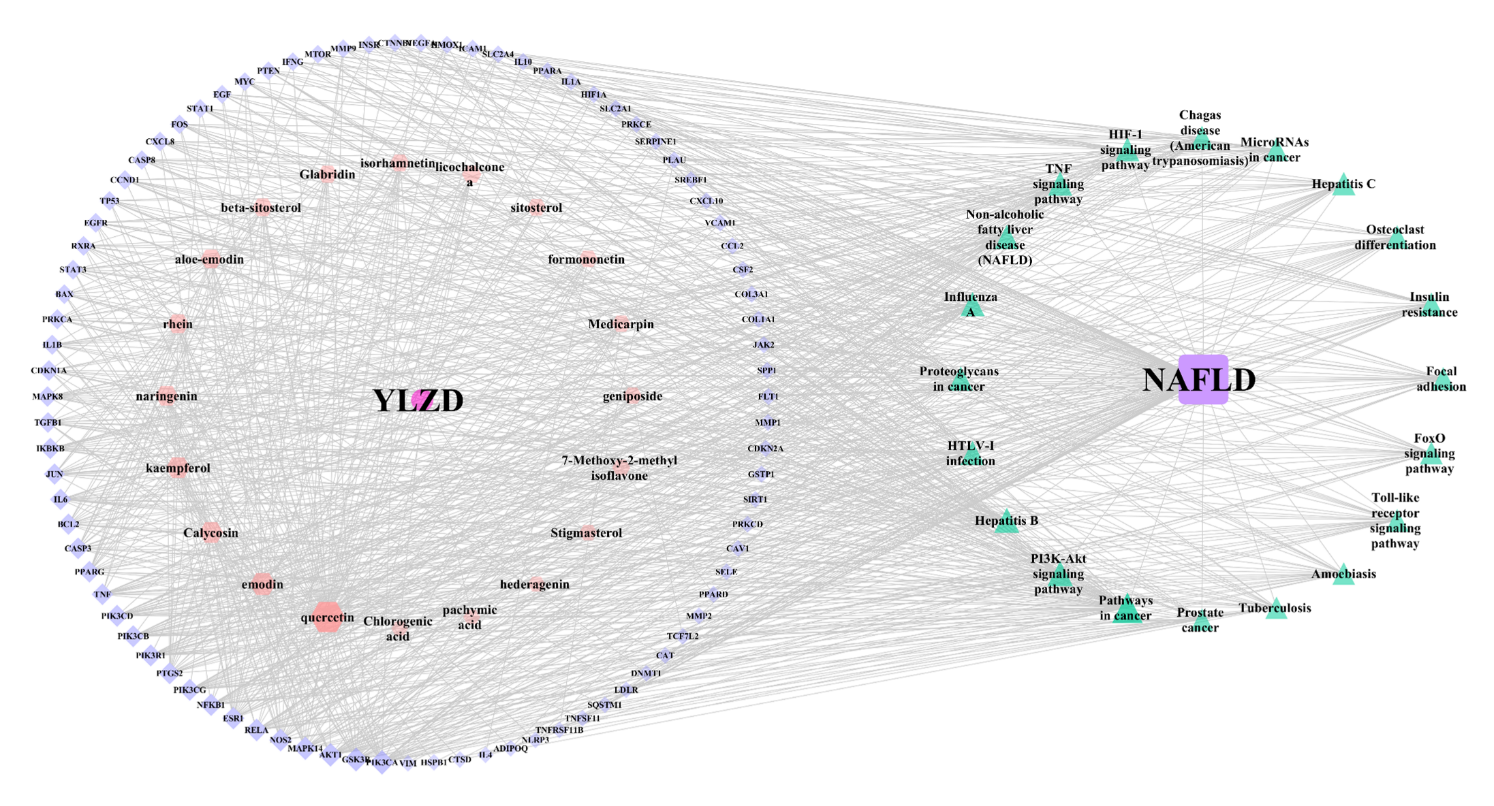

Supplement: Supplementary file 1 [file DataSheet1.zip › Supplementary materials/Supplementary Figures.docx]
